# Detection and molecular characterization of major enteric pathogens in calves in central Ethiopia

**DOI:** 10.1186/s12917-024-04258-7

**Published:** 2024-09-04

**Authors:** Julia Bergholm, Tesfaye Sisay Tessema, Anne-Lie Blomström, Mikael Berg

**Affiliations:** 1 Department of Animal Biosciences, Swedish University of Agricultural Sciences, Uppsala, Sweden; 2 Institute of Biotechnology, Addis Ababa University, Addis Ababa, Ethiopia

**Keywords:** Ethiopia, Calf diarrhea, *Cryptosporidium*, *E. coli* K99 +, Rotavirus A, Bovine coronavirus

## Abstract

**Background:**

Calf diarrhea is a major cause of morbidity and mortality in the livestock sector worldwide and it can be caused by multiple infectious agents. In Ethiopia, cattle are the most economically important species within the livestock sector, but at the same time the young animals suffer from high rates of morbidity and mortality due to calf diarrhea. However, studies including both screening and molecular characterization of bovine enteric pathogens are lacking. Therefore, we aimed to both detect and molecularly characterize four of the major enteric pathogens in calf diarrhea, *Enterotoxigenic Escherichia coli (E. coli* K99 +), *Cryptosporidium* spp*.*, rotavirus A (RVA), and bovine coronavirus (BCoV) in calves from central Ethiopia. Diarrheic and non-diarrheic calves were included in the study and fecal samples were analyzed with antigen-ELISA and quantitative real-time PCR (qPCR). Positive samples were further characterized by genotyping PCRs.

**Results:**

All four pathogens were detected in both diarrheic and non-diarrheic calves using qPCR and further characterization showed the presence of three *Cryptosporidium* species, *C. andersoni*, *C. bovis* and *C. ryanae*. Furthermore, genotyping of RVA-positive samples found a common bovine genotype G10P[11], as well as a more unusual G-type, G24. To our knowledge this is the first detection of the G24 RVA genotype in Ethiopia as well as in Africa. Lastly, investigation of the spike gene revealed two distinct BCoV strains, one classical BCoV strain and one bovine-like CoV strain.

**Conclusions:**

Our results show that *Cryptosporidium* spp*.*, *E. coli* K99 + , RVA and BCoV circulate in calves from central Ethiopia. Furthermore, our findings of the rare RVA G-type G24 and a bovine-like CoV demonstrates the importance of genetic characterization.

**Supplementary Information:**

The online version contains supplementary material available at 10.1186/s12917-024-04258-7.

## Background

Ethiopia is situated in eastern Africa and is home to the largest livestock population on the continent. Livestock plays a key role in Ethiopia’s agricultural production and cattle are considered the most economically important species, with over 12 million of the 14 million livestock-holding households owning at least one cattle [1, 2]. However, high morbidity and mortality, especially among young animals, results in large losses in the livestock sector [3]. This impacts both animal health and the people depending on livestock for their livelihood. Diarrheic disease has been reported to be a leading cause of mortality and morbidity in neonatal calves all over Ethiopia [4–7].

Neonatal calf diarrhea is a multifactorial disease and calves are especially susceptible to diarrheic disease during their first weeks of life, when the immune system is not yet fully developed. Diarrheic disease in calves can be caused by multiple bacterial, protozoan, and viral agents, but some are considered major enteric pathogens. Four of the major enteric agents include *Enterotoxigenic Escherichia coli (E. coli* K99 +), *Cryptosporidium* spp*.*, rotavirus, and coronavirus [8]. *E. coli* K99^+^ is a gram-negative bacterium that primarily causes diarrhea within the first days of the neonatal calf’s life. It attaches and colonizes the intestinal epithelium and secretes heat-labile enterotoxin, causing diarrhea [9, 10]. *Cryptosporidium* is a unicellular protozoan that causes enteric disease in a variety of mammals, including humans. Four species of *Cryptosporidium* are commonly found in cattle: *C. parvum*, *C. bovis*, *C. ryanae* and *C. andersoni.* However, mainly *C. parvum* is associated with clinical disease in neonatal calves [11]. Rotaviruses are double-stranded RNA viruses that cause enteric disease in both humans and animals. In calves, rotavirus type A (RVA) is the most common subtype, primarily causing diarrhea within the first 14 days of life [8]. RVAs can be classified based on the two outer capsid proteins, the surface glycoprotein encoded by the VP7 gene (G-type), and the protease-sensitive attachment protein encoded by the VP4 gene (P-type) [12]. Bovine coronavirus (BCoV) is a single-stranded RNA virus that belongs to the species *Betacoronavirus 1*. BCoV is associated with diarrhea in neonatal calves and winter dysentery in adult cattle. Furthermore, BCoV can infect the respiratory tract, causing respiratory disease in cattle of all ages [13]. The S1 subunit of the spike gene is variable and has been utilized for molecular epidemiology of BCoV strains [14, 15].

All four enteric pathogens have been detected at different prevalence rates in calves in Ethiopia [16–19]. However, studies including molecular characterization of the pathogens are lacking. Therefore, the aim of this study was to investigate and characterize the genotypes of *Cryptosporidium* spp*.*, *E. coli* K99 + , RVA and BCoV circulating in calves in central Ethiopia.

## Results

### Detection of enteric pathogens with antigen-ELISA and qPCR

A total of 47 calves (< 2 months of age) were sampled in four different towns in central Ethiopia. Antigen-ELISA (Ag-ELISA) was used to screen 44 of the fecal samples for *E. coli* K99 + , *Cryptosporidium* spp*.*, RVA and BCoV. Following analysis with Ag-ELISA, all 47 samples were screened for the same enteric pathogens using quantitative real-time (qPCR) (Table 1). When using qPCR, a higher proportion of positive samples was observed for all four tested pathogens in comparison to Ag-ELISA. *Cryptosporidium* spp. was the most common pathogen identified in all calves followed by RVA when analyzed with qPCR, with 72.3% and 19.1% of samples being positive for each pathogen, respectively. When using Ag-ELISA the positivity rate was 4.5% for *Cryptosporidium* spp. and 2.3% for RVA. qPCR and Ag-ELISA detected *E. coli* K99 + at the rates of 10.6% and 4.5%, respectively. Lastly, 8.5% of the calves were positive for BCoV with qPCR and no calves tested positive for BCoV with the Ag-ELISA kit.

**Table 1:** The frequencies of the four enteric pathogens using Ag-ELISA and qPCR

Pathogen	Number of positive samples (%)	
	**Ag-ELISA**	**qPCR**
*E. coli* K99 +	2/44 (4.5)	5/47 (10.6)
*Cryptosporidium* spp.	2/44 (4.5)	34/47 (72.3)
RVA	1/44 (2.3)	9/47 (19.1)
BCoV	0/44 (0.0)	4/47 (8.5)

**Fig. 1:**
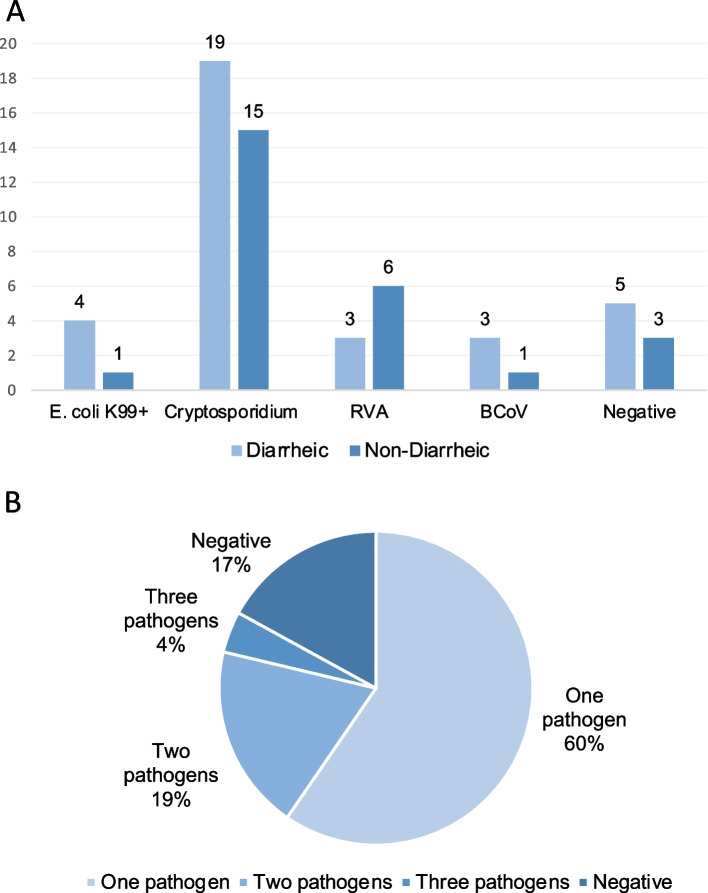
qPCR based detection rates of each pathogen based on diarrheal status. Numbers above bars indicate number of qPCR positive animals (**A**). Percentage of calves testing positive for one, two, three or no pathogens with qPCR (**B**)

*E. coli* K99 + , *Cryptosporidium* spp*.*, RVA and BCoV were found in both diarrheic and non-diarrheic calves. Eight of the calves tested negative for all four enteric pathogens when screened with qPCR. Of the calves that tested negative five were diarrheic and three non-diarrheic (Fig. 1A). Of the positive calves, the majority tested positive for one pathogen (60%, 28/47), followed by two pathogens (19%, 9/47), and lastly three pathogens (4%, 2/47) (Fig. 1B). Both diarrheic and non-diarrheic calves tested positive for one and two pathogens. The two calves that tested positive for three pathogens were both diarrheic (data not shown).

### *Cryptosporidium* species

All 34 qPCR *Cryptosporidium*-positive samples generated the expected 18S rRNA gene products in nested PCR. Three species of *Cryptosporidium* were identified, *C. andersoni*, *C. bovis* and *C. ryanae*. *C. andersoni* was the most common species (62%, 21/34), followed by *C. bovis* (21%, 7/34) and *C. ryanae* (15%, 5/34). One sample that was positive using qPCR was not possible to genotype. Interestingly, no *C. parvum* was detected in any of the positive samples. Furthermore, the two specimens that tested positive for *Cryptosporidium* with Ag-ELISA were molecularly characterized as *C. andersoni.*

### G- and P-typing of RVA

To determine G- and P-genotypes of RVA-positive samples partial fragments of the VP7 and VP4 gene were amplified. G-type was determined for nine RVA-positive samples, generating the expected VP7 gene products after nested PCR. Two different G-types were detected, G10 in eight samples, and G24 in one sample. Characterization of the VP4 gene found one P-type, P[11]. However, P-typing was not possible for eight of the RVA-positive samples. In total, three genotypes were found, G10P[11] (1/9), G24P[x] (1/9) and G10P[x] (7/9). The G24P[x] strain was isolated from a calf on a farm in Sebeta and the remaining samples genotyped as G10P[11] and G10P[x] originated from three different farms in Holeta.

**Fig. 2:**
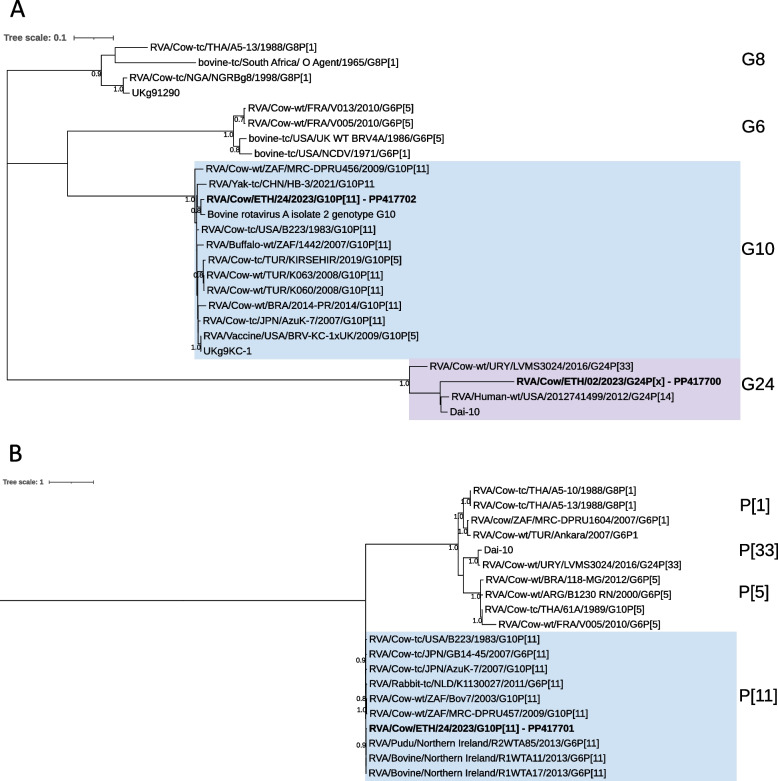
Maximum likelihood trees of the VP7 gene (**A**) and partial VP4 gene (nt 11–887, VP8* segment) (**B**) rooted at the midpoint. G10 and P[11] genotypes are highlighted with a blue background and G24 genotypes with a purple background. Strains identified in the current study are indicated by bold text. Bootstrap values > 0.7 are displayed

Phylogenetic analysis was performed on the VP7 gene of the Ethiopian G10P[11] and G24P[x] strains. For VP4 analysis only G10P[11] was included. Phylogenetic analysis revealed that the VP7 genes of G10P[11] and G24P[x] grouped with sequences from the respective genotypes, G10 and G24 (Fig. 2A). A similar tree structure was observed for the VP4 gene of G10P[11], clustering with other P[11] isolates (Fig. 2B).

### Molecular characterization of the S1 subunit of BCoV

**Fig. 3:**
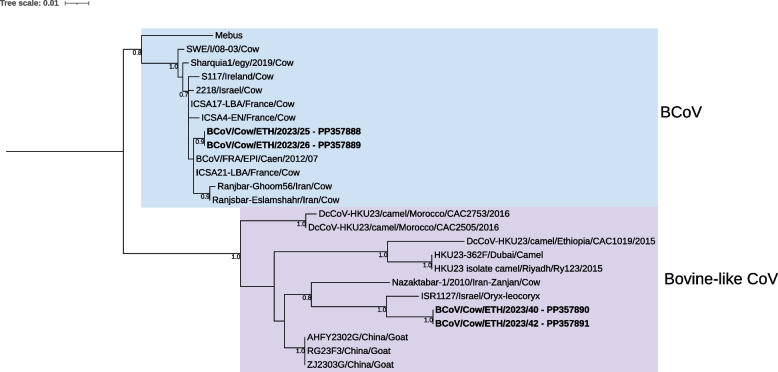
Maximum likelihood tree of the hypervariable region of the S1 subunit (nt: 1332–1778 of Mebus, U00735) rooted at the midpoint. Classical BCoV strains are highlighted with a blue background and bovine-like CoV strains with a purple background. Strains identified in the current study are indicated by bold text. Bootstrap values > 0.7 are displayed

Four samples tested positive for BCoV in qPCR and were successfully genotyped by amplification and sequencing of the hypervariable region of the S1 subunit. BCoV/Cow/ETH/2023/25 and BCoV/Cow/ETH/2023/26 were isolated from the same farm in Holeta. BLASTn search identified BCoV strains isolated from cattle in France as closest relatives, with > 99% nucleotide identity. The other two samples, BCoV/Cow/ETH/2023/40 and BCoV/Cow/ETH/2023/42 were isolated from the same farm in Sululta. Interestingly, the most similar sequence identified with BLASTn with 96.9% identical nucleotides, was a bovine-like coronavirus (CoV) isolated from a white oryx (*Oryx leucoryx*) in Israel. Phylogenetic analysis of the S1 hypervariable region revealed that samples BCoV/Cow/ETH/2023/25 and BCoV/Cow/ETH/2023/26 clustered with classical BCoV strains isolated from cattle in different countries. As indicated by the BLASTn search, BCoV/Cow/ETH/2023/40 and BCoV/Cow/ETH/2023/42 grouped with bovine-like CoVs isolated from different animal species instead of classical BCoV (Fig. 3).

### Discussion

The present study identified and molecularly characterized *Cryptosporidium*, *E. coli* K99 + , RVA and BCoV in calves from central Ethiopia and all four pathogens were detected using qPCR. Furthermore, the four major enteric pathogens were found in fecal samples from both diarrheic and non-diarrheic calves. Similar findings have been observed in a previous study conducted in Ethiopia [20], as well as in reports from other countries [21, 22]. All four pathogens are causative agents of neonatal calf diarrhea; however, the clinical picture can differ depending on immune status of the animal and at what stage of the infection the sample is taken. A majority of the screened calves tested positive for one or more pathogens, however, eight calves tested negative for both *Cryptosporidium*, *E. coli* K99 + , RVA and BCoV. Five of the calves testing negative had active diarrhea during sampling, indicating that other pathogens, not tested for in this study, could be the causative agents of the disease. Screening for additional enteric pathogens or metagenomics-based analysis of the samples could provide insight on other microbes possibly involved in the disease.

When screening the samples with the commercial Ag-ELISA kit fewer samples tested positive compared to qPCR and no BCoV could be identified using the Ag-ELISA. Three out of the four samples that were positive in both qPCR and Ag-ELISA had lower ct values (ct < 30). Together, this indicates a lower sensitivity of the commercial Ag-ELISA or difference in surface antigenicity between the pathogens detected by the kit and the pathogens circulating in the country. Ag-ELISAs are often less sensitive than qPCR [8, 23] but can provide a preliminary overview of what pathogens are circulating in animals when access to laboratory facilities with molecular techniques is limited.

qPCR detected *E. coli* K99 + at the rate of 10.6% (5/47) in calves from Holeta and Bishoftu. Being a small sample size, no conclusions can be drawn on the true prevalence rate of *E. coli* K99 + in the calf population. However, similar detection rates have been seen for *E. coli* K99 + in previous studies on calves in Ethiopia, reporting *E. coli* K99 + in 11.1–22.1% of sampled calves [17, 19].

*Cryptosporidium* spp*.* was the most commonly detected enteric pathogen using qPCR. Genotyping using the partial 18S rRNA gene showed three different species among the qPCR positive calves, *C. andersoni*, *C. bovis* and *C. ryanae.* One sample was un-typeable even after repeated trials of sequencing as well as cloning. Most likely there was an insufficient amount of genetic material available for molecular characterization as the sample had a high ct-value in the qPCR (data not shown). Interestingly, no samples were genotyped as *C. parvum,* which is believed to be the most predominant *Cryptosporidium* species among neonatal calves, while infection with *C. andersoni*, *C. bovis* and *C. ryanae* is mostly associated with older calves [11]. However, our finding correlates with another study from central Ethiopia which screened over 400 calves below five months of age. The authors detected *C. andersoni*, *C. bovis* and *C. ryanae* but no *C. parvum*, suggesting that *C. parvum* could have a low prevalence in calves in the central region of Ethiopia [24]. Moreover, a study from 2019 in Sweden investigated the infection dynamics of *C. bovis* and *C. ryanae* in dairy herds free from *C. parvum* infection. The study found that *C. bovis* and *C. ryanae* infections were most prevalent in calves aged 4–5 weeks, which is earlier than reported in other studies, indicating that the absence of *C. parvum* affects the infection dynamics of *C. bovis* and *C. ryanae* [25]. This provides a possible explanation to the previous and current findings of *C. andersoni*, *C. bovis* and *C. ryanae* in young calves in the central region of Ethiopia.

Genotyping of the nine RVA-positive samples found two G-types, G10 and G24 and one P-type, P[11]. To our knowledge this is the first genotyping of bovine RVAs in Ethiopia. However, a majority of the RVA positive samples could not be P-typed, which is most likely explained by few viral RNA copies being available in these samples, all having ct values > 30 in the qPCR (data not shown). G10[P11] is an RVA genotype commonly found in cattle [26] and the genotype identified in the current study clustered with other G10 and P[11] sequences isolated from different countries. More unusual is the G24P[x] strain identified in a calf from Sebeta, a G-type which has only been detected at three occasions prior to this study, twice in cattle from Japan and Uruguay (G24P[33]) [27, 28], and once in a human in USA (G24P[14] genotype) [29]. Phylogenetic analysis revealed that the G24 sequence from the current study grouped with the isolated G24P[33] strains from Japan and Uruguay as well as the human G24P[14] strain. To our knowledge, this is the first detection of the G24 G-type in Ethiopia, as well as in Africa. Being segmented viruses, RVAs are capable of reassortment and interspecies transmission, and the G24P[33] and G24P[14] strains are hypothesized to have arisen from reassortment between RVAs from different species. Further characterization of the remaining segments by high-throughput sequencing could reveal information on the origin and possible reassortment events of the identified G24P[x] strain.

BCoV was identified in four calves from two different farms, one in Holeta and one in Sululta. Molecular characterization of the spike gene revealed that the two BCoV isolates from Holeta (BCoV/Cow/ETH/2023/25 and BCoV/Cow/ETH/2023/26) belonged to classical BcoV, clustering with other BCoV isolated from cattle from different countries. However, the two isolates from the farm in Sululta (BCoV/Cow/ETH/2023/40 and BCoV/Cow/ETH/2023/42), differed from the classical BCoV strains and grouped with bovine-like CoVs isolated from different animals. Furthermore, a Dromedary camel CoV-HKU23 (DcCoV-HKU23) isolated from a dromedary camel in Ethiopia in 2015 [30] clustered with BCoV/Cow/ETH/2023/40 and BCoV/Cow/ETH/2023/42, demonstrating that the strain identified in the two calves from Sululta is more similar to the Ethiopian DcCoV-HKU23 than to the classical BCoV strain isolated from the two calves in the nearby town, Holeta. BCoV and bovine-like CoVs are genetically similar and belong to the same species, *Betacoronavirus 1*, and are known to be able to cross the species barrier [13]. This suggests that either the calves in Sululta have been infected with a bovine-like CoV, either from contact with dromedary camels or other ruminants, or that a BCoV originating from bovine-like CoVs circulates among calves together with classical BCoV strains. Sequencing of the full BCoV and bovine-like CoV genomes could provide a better picture of the evolution and common ancestors, as well as potential recombination events between different strains.

This study has some limitations that need to be addressed. First is the small sample size, which resulted from a limited study time and restrictions in the field. Secondly, both the geographical area and study time of two months prevent any conclusions from being drawn on the seasonality of the pathogens or their geographical spread in Ethiopia. However, with molecular characterization of pathogens in calf diarrhea lacking in Ethiopia, this study provides important insight into circulating species and genotypes. The results presented here can hopefully aid and promote future studies on the subject in Ethiopia, involving more animals and additional regions.

## Conclusions

In summary, we detected *Cryptosporidium* spp*.*, *E. coli* K99 + , RVA and BCoV in calves from central Ethiopia. *Cryptosporidium*, RVA and BCoV were further characterized to determine species and genotypes and to our knowledge this is the first molecular characterization of bovine RVAs and BCoV in Ethiopia. Our findings of the rare RVA G-type G24P[x] and a bovine-like CoV demonstrates the importance of genetic characterization in combination with conventional screening. It enables the discovery of novel or uncommon variants of different pathogens that would most likely remain undetected with normal screening methods. This knowledge is key to detect emerging strains that can affect the animal health or have possible zoonotic potential, as well as providing better vaccination strategies and treatment options.

## Methods

### Study area and sample collection

**Fig. 4:**
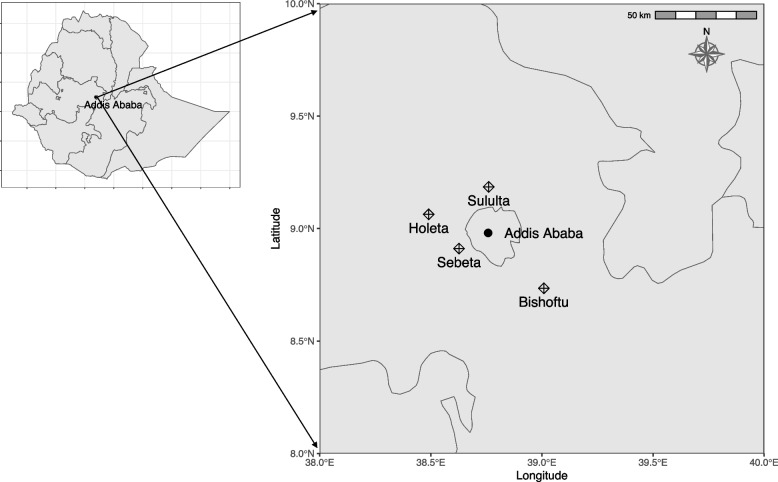
Map showing the location of the towns in Ethiopia where sampling was conducted. The towns Sebeta, Holeta, Sululta and Bishoftu are represented by diamonds. The capital Addis Ababa is represented by a black circle. Maps were generated using R software (version 4.2.2)

The study was conducted in four small towns, Sebeta, Holeta, Sululta and Bishoftu, outside the capital Addis Ababa in central Ethiopia during January and February 2023 (Fig. 4). A total of 47 calves (diarrheic = 26, non-diarrheic = 21) up to two months of age from 16 different farms were included in the study using purposive sampling. A trained veterinarian sampled all calves included in the study and determined if they were diarrheic or non-diarrheic. Fecal samples were collected directly from the rectum in sterile stool cups and transported under cold chain from the field to the Institute of Biotechnology, AAU, Addis Ababa, where the samples were stored at -20 °C until further processing. Information on health status and age of each sampled calf was retrieved from the animal owner using a questionnaire form (Supplementary data).

### Sample preparation

All fecal samples were divided into three aliquots. For DNA and RNA extraction two fecal aliquots were suspended in DNA/RNA Shield™ (10% w/v) and homogenized using ZR BashingBead™ Lysis Tubes (Zymo research, Irvine, CA, USA) and a Vortex-Genie in combination with a horizontal tube holder (Thermo Fisher Scientific, Waltham, MA, USA). The homogenization was done according to manufacturer’s instructions for lower speed bead beating devices. The homogenates were centrifuged at 12,000 × *g* for 1 min and the supernatant was collected for future analysis. Homogenates to be used for RNA extraction were further processed by filtering with ultracentrifuge membrane filters (0.45 μM; Utrafree®-MC centrifugal Filter, Darmstadt, Germany) at 12,000 × *g* for 4 min. Lastly, one aliquot was kept untreated for analysis with Ag-ELISA.

### Antigen-ELISA

The untreated fecal samples were screened for the presence of *E. coli* K99 + , *Cryptosporidium* spp*.*, RVA and BCoV using the commercial Ag-ELISA kit BIO K 151 (Bio-X Diagnostics, Belgium) following manufacturer’s instructions. After adding stop solution, the optical densities were measured at 450 nm using a Multiskan™ FC Microplate Photometer (Thermo Fisher Scientific, Waltham, MA, USA). Due to limited access to Ag-ELISA kits three non-diarrheic samples were excluded from the analysis.

### Nucleic acid extraction and cDNA synthesis

DNA was extracted using the Quick-DNA™ Fecal/Soil Microbe Miniprep Kit (Zymo research, Irvine, CA, USA) according to manufacturer´s instructions. The DNA was eluted in 100 µL of DNA elution buffer and stored at -80 °C until further use. Total RNA was extracted using a combination of TRIzol (Invitrogen, Carlsbad, CA, USA) and GeneJet RNA extraction columns (Thermo Fisher Scientific, Waltham, MA, USA). In short, an equal volume of TRIzol was added to the filtered samples and mixed. Following addition of chloroform, the aqueous phase was diluted with an equal volume of 70% ethanol and purified with GeneJet columns. The RNA was eluted in 40 µL of nuclease-free water and stored at -80 °C until further use. cDNA synthesis was performed on the extracted RNA using SuperScript III reverse transcriptase (Invitrogen, Carlsbad, CA, USA) with 5 µL of RNA template, according to manufacturer’s instructions.

### qPCR

qPCR for the detection of *E. coli* K99 + , *Cryptosporidium* spp., RVA and BCoV was performed using a 20 µL iTaq Universal Probes Supermix reaction with 300–600 nM primers, 100–200 nM probe and 2 µL of template. Each reaction was run as singleplex with pathogen-specific primers (Table 2) on a C1000 Touch Thermal Cycler™ (Bio-Rad, Hercules, CA, USA). Positive and negative controls were included in all qPCR runs. Number of cycles and cut-off values for each qPCR is provided in supplementary table S1.

**Table 2:** List of primers and probes used for detection of each enteric pathogen in qPCR

Pathogen	Primer/probe sequence (5’-3’)	Gene	Reference
*E. coli* K99 +	Fw: GCTATTAGTGGTCATGGCACTGTAG Rv: TTTGTTTTCGCTAGGCAGTCATTA Pb: FAM-ATTTTAAACTAAAACCAGCGCCCGGCA-BHQ1	K99	[31, 32]
*Cryptosporidium* spp*.*	Fw: GGTTGTATTTATTAGATAAAGAAC Rv: TAGGCCAATACCCTACCGTC Pb: FAM-CATATCATTCAAGTTTCTGACCTATC-MGB^a^	18S rRNA	[33]
RVA	Fw: TGATTCTGCTTCAAACGATCCA Rv: GCATTTGTCTTAACTGCATTCGA Pb: VIC-TCACCAGCTTTTCGATAAG-MGB	NSp5	[34]
BCoV	Fw: CTGGAAGTTGGTGGAGTT Rv: ATTATCGGCCTAACATACATC Pb: FAM -CCTTCATATCTATACACATCAAGTTGTT-BHQ1	M	[35]

^a^Probe sequence modified from Stroup et al

### Genotyping PCRs and sequencing

To determine *Cryptosporidium* species, a nested PCR was utilized to amplify an 830 bp fragment of the 18S rRNA gene, using primers 5′-TTC TAG AGC TAA TAC ATG CG-3′ and 5′-CCC ATT TCC TTC GAA ACA GGA-′3 in the primary PCR and 5´-GGA AGG GTT GTA TTT ATT AGA TAA AG-3′ and 5´-AAG GAG TAA GGA ACA ACC TCC A-3′ in the secondary PCR [36, 37].

All samples that tested positive for RVA in the qPCR were subjected to nested PCRs to determine G-type (VP7) and P-type (VP4). For VP7, primers Gra-5 and Gra-3 were used [38] together with primers VP7-up2 and VP7-down3 [39] generating a 956 bp product. If no product was visible a second PCR designed for RVA samples with a lower viral load was applied. Primers N-VP7F1 and N-VP7R1 were used in the primary reaction, followed by a secondary reaction with primers N-VP7F2 and N-VP7R2, amplifying a 193 bp product [40]. For partial amplification of the VP4 gene, primers Con-3 and Con-2 were used [41], generating an 850 bp product. If amplification was unsuccessful with primers Con-3 and Con-2, the protocol by Mijatovic-Rustempasic *et. al* with primers N-VP4F1 and N-VP4R1 in the primary PCR and primers N-VP4F2 and N-VP4R2 in the secondary PCR was used [40].

Molecular characterization of BCoV was performed by amplifying the hypervariable region of S1 subunit of the spike gene. Primers S1HS and S1HA were used in the first reaction and primers S1NS and S1NA in the second reaction, amplifying a 488 bp product [15]. Modified primers were used on BCoV-positive samples that did not generate any product using the original nested PCR. In the new reaction primer S1HS together with S1HA**-**mod (5´-C**A**GA**T**ACACGACCACTAT-3´) was used, followed by primers S1NS**-**mod (5´-GTTTCTGTTAGCAG**A**TT**A**AA-3´) and S1NA**-**mod (5´-ATATTACACCT**G**TC**T**CCTTG-3´). Modified bases in the primer sequences are indicated by bold text.

All PCR reactions were run for 35 cycles in a 25 µL reaction with Invitrogen™ Platinum Superfi DNA Polymerase (Invitrogen, Carlsbad, CA, USA), 0.5 µM of each primer and 2 µL of template according to manufacturer’s instructions. PCR products were visualized on 1–2% agarose gels, depending on product size, stained with GelRed. Gels were run at 100 V for 60 min and purified using the GeneJET™ gel extraction kit (Thermo Fisher Scientific, Waltham, MA, USA). Purified products were sent for sequencing at Macrogen Europe. If sequencing failed or was of poor quality the PCR product was cloned into the pJET1.2/blunt vector using the CloneJET PCR Cloning Kit (Thermo Fisher Scientific, Waltham, MA, USA) and transformed into Dh5α competent cells. Plasmid DNA was subsequently purified using the GeneJET™ Plasmid Miniprep Kit (Thermo Fisher Scientific, Waltham, MA, USA) and sent for re-sequencing.

### Sequence analysis and phylogenetics

Forward and reverse sequences were assembled and manually curated in Geneious prime (version 2023.2.1). Consensus sequences were compared to nucleotide reference sequences using BLASTn (Basic Local Alignment Search Tool, NCBI, http://www.ncbi.nlm.nih.gov/blast/Blast.cgi) to determine genotypes. Representative sequences were deposited into NCBI GenBank under accession numbers PP358250-52 (*Cryptosporidium*), PP417700-02 (RVA) and PP357888-91 (BCoV).

Reference sequences (supplementary tables S2-S4) were downloaded from GenBank and aligned with sequences from the study in Geneious prime (version 2023.2.1) using MUSCLE [42]. Aligned sequences were imported into MEGA X [43] and phylogenetic trees were constructed using the maximum likelihood method with 1,000 bootstrap replicates. The best fitting substitution model was determined for each dataset by lowest BIC score. The phylogenetic trees were visualized in Interactive Tree Of Life (iTOL) version 6.8.1 [44].

## Supplementary Information

Supplementary Material 1.

Supplementary Material 2.

## Data Availability

Representative nucleotide sequences are available at NCBI GenBank under accession numbers PP358250-52 (*Cryptosporidium*), PP417700-02 (RVA) and PP357888-91 (BCoV). The remaining datasets used and analyzed during the current study are available from the corresponding author on reasonable request.
