# Supplementary material for: Detection and molecular characterization of major enteric pathogens in calves in central Ethiopia
Source: BMC Vet Res. 2024 Sep 4;20:389. doi: 10.1186/s12917-024-04258-7 (PMC11373192; doi:10.1186/s12917-024-04258-7)
Supplement: Supplementary file 1 — Supplementary Material 1. [file 12917_2024_4258_MOESM1_ESM.pdf]

Date:

**Farm ID:**	**Animal info**			**Symptoms**			**Antibiotic treatment**		**Other comments**
**Sample ID**	Breed	Age	Sex	Diarrhea	Healthy	Description of feces	Yes	No	
