# Supplementary material for: Detection and molecular characterization of major enteric pathogens in calves in central Ethiopia
Source: BMC Vet Res. 2024 Sep 4;20:389. doi: 10.1186/s12917-024-04258-7 (PMC11373192; doi:10.1186/s12917-024-04258-7)
Supplement: Supplementary file 2 — Supplementary Material 2. [file 12917_2024_4258_MOESM2_ESM.docx]

**Supplementary Data**

**Table S1.** Cycling and cut-off values for qPCRs.

| **Pathogen** | **Cycles** | **Cut-off** | **Comment** |
| --- | --- | --- | --- |
| *E. coli* K99+ | 40 | Positive: <35 |  |
| *Cryptosporidium* | 48 | Positive: <45 |  |
| RVA | 40 | Positive: <36  Intermediate: 36<40 | Samples with intermediate ct-values were considered positive if they generated a PCR product in the RVA genotyping PCR. |
| BCoV | 45 | Positive: <40 |  |

| **Table S2.** Reference sequences VP7 (RVA) |  |  |  |
| --- | --- | --- | --- |
| **Strain** | **Country** | **Animal** | **GenBank accession no.** |
| RVA/Cow-tc/USA/B223/1983/G10P[11] | USA | Cow | LC133552 |
| RVA/Vaccine/USA/BRV-KC-1xUK/2009/G10P[5] | USA | Cow | KC215545 |
| RVA/Cow-tc/JPN/AzuK-7/2007/G10P[11] | Japan | Cow | LC553633 |
| RVA/Cow-wt/TUR/K063/2008/G10P[11] | Turkey | Cow | MZ912999 |
| RVA/Cow-wt/TUR/K060/2008/G10P[11] | Turkey | Cow | MZ913000 |
| RVA/Buffalo-wt/ZAF/1442/2007/G10P[11] | South Africa | Buffalo | MT234356 |
| RVA/Cow-wt/ZAF/MRC-DPRU456/2009/G10P[11] | South Africa | Cow | MW771173 |
| RVA/Yak-tc/CHN/HB-3/2021/G10P11 | China | Yak | ON711389 |
| RVA/Cow-wt/BRA/2014-PR/2014/G10P[11] | Brazil | Cow | MG269492 |
| KC-1xUK reassortant (UKg9KC-1) | USA | Cow | HQ844013 |
| RVA/Cow-tc/TUR/KIRSEHIR/2019/G10P[5] | Türkiye | Cow | OQ082576 |
| Bovine rotavirus A isolate 2 genotype G10 | Egypt | Cow | MW751820 |
| RVA/Cow-xx/CHN/DQ-75/2008/G10P[11] | China | Cow | GU144587 |
| RVA/Human-wt/USA/2012741499/2012/G24P[14] | USA | Human | KT281128 |
| Dai-10 | Japan | Cow | AB513837 |
| RVA/Cow-wt/URY/LVMS3024/2016/G24P[33] | Uruguay | Cow | MN649717 |
| RVA/Cow-wt/FRA/V005/2010/G6P[5] | France | Cow | HE646640 |
| RVA/Cow-wt/FRA/V013/2010/G6P[5] | France | Cow | HE646641 |
| bovine-tc/USA/NCDV/1971/G6P[1] | USA | Cow | JF693034 |
| bovine-tc/USA/UK WT BRV4A/1986/G6P[5] | USA | Cow | JF693067 |
| 1290xUK reassortant (UKg91290) | USA | Cow | GQ225781 |
| bovine-tc/South Africa/'O' Agent/1965/G8P[1] | South Africa | Cow | JF693045 |
| RVA/Cow-tc/NGA/NGRBg8/1998/G8P[1] | Nigeria | Cow | LC119109 |
| RVA/Cow-tc/THA/A5-13/1988/G8P[1] | Thailand | Cow | LC133530 |

| **Table S3.** Reference sequences VP4 (RVA) |  |  |  |
| --- | --- | --- | --- |
| **Strain** | **Country** | **Animal** | **GenBank accession no.** |
| RVA/Bovine/Northern Ireland/R1WTA11/2013/G6P[11] | Ireland | Cow | OL988935 |
| RVA/Pudu/Northern Ireland/R2WTA85/2013/G6P[11] | Ireland | Pudu | OL989019 |
| RVA/Bovine/Northern Ireland/R1WTA17/2013/G6P[11] | Ireland | Cow | OL988983 |
| RVA/Rabbit-tc/NLD/K1130027/2011/G6P[11] | Netherlands | Rabbit | KC488888 |
| RVA/Cow-tc/JPN/GB14-45/2007/G6P[11] | Japan | Cow | LC553609 |
| RVA/Cow-wt/ZAF/Bov7/2003/G10P[11] | South Africa | Cow | MW771141 |
| RVA/Cow-wt/ZAF/MRC-DPRU457/2009/G10P[11] | South Africa | Cow | MW771185 |
| RVA/Cow-tc/JPN/AzuK-7/2007/G10P[11] | Japan | Cow | LC553631 |
| RVA/Cow-tc/THA/A5-10/1988/G8P[1] | Thailand | Cow | LC133517 |
| RVA/Cow-tc/THA/A5-13/1988/G8P[1] | Thailand | Cow | LC133528 |
| RVA/cow/ZAF/MRC-DPRU1604/2007/G6P[1] | South Africa | Cow | KF636259 |
| RVA/Cow-wt/TUR/Ankara/2007/G6P1 | Türkiye | Cow | JX076838 |
| Dai-10 | Japan | Cow | AB513836 |
| RVA/Cow-wt/URY/LVMS3024/2016/G24P[33] | Uruguay | Cow | MN649674 |
| RVA/Cow-wt/BRA/118-MG/2012/G6P[5] | Brazil | Cow | MG269509 |
| RVA/Cow-wt/ARG/B1230_RN/2000/G6P[5] | Argentina | Cow | KC895834 |
| RVA/Cow-wt/FRA/V005/2010/G6P[5] | France | Cow | HE646659 |
| RVA/Cow-tc/THA/61A/1989/G10P[5] | Thailand | Cow | LC133539 |

| **Table S4.** Reference sequences Spike (BCoV) |  |  |  |
| --- | --- | --- | --- |
| **Strain** | **Country** | **Animal** | **GenBank accession no.** |
| ICSA17-LBA | France | Cow | MG757144 |
| BCoV/FRA/EPI/Caen/2012/07 | France | Cow | KT318117 |
| ICSA4-EN | France | Cow | MG757143 |
| ICSA21-LBA | France | Cow | MG757138 |
| 2218 | Israel | Cow | MW310535 |
| Ranjbar-Ghoom56 | Iran | Cow | MK932865 |
| Ranjbar-Eslamshahr | Iran | Cow | MK932864 |
| S117 | Ireland | Cow | OR271256 |
| Sharquia1/Egy/2019 | Egypt | Cow | MN531697 |
| SWE/I/08-3 | Sweden | Cow | KF169933 |
| AHFY2302G | China | Goat | OR077309 |
| RG23F3 | China | Goat | OR077308 |
| ZJ2303G | China | Goat | OR077307 |
| ISR1127 | Israel | Oryx leocoryx | OM397541 |
| DcCoV-HKU23/camel/Ethiopia/CAC1019/2015 | Ethiopia | Camel | MN514962 |
| DcCoV-HKU23/camel/Morocco/CAC2753/2016 | Morocco | Camel | MN514970 |
| DcCoV-HKU23/camel/Morocco/CAC2505/2016 | Morocco | Camel | MN514971 |
| HKU23 isolate camel/Riyadh/Ry123/2015 | Saudi Arabia | Camel | KT368891 |
| HKU23 strain HKU23-362F | Dubai | Camel | MN514971 |
| Nazaktabar-1/2010/Iran-Zanjan | Iran | Cow | MH371012 |
| Mebus | USA | Cow | U00735 |
